# Multi-Strain Probiotic and Bee Pollen Supplementation Attenuates CCl_4_-Induced Altered Intestinal Tight Junctions in Rodents

**DOI:** 10.3390/cimb48030310

**Published:** 2026-03-13

**Authors:** Nada Alsayari, Ramesa Shafi Bhat, Seema Zargar, Abeer M. Aldbass, Sooad Al-Daihan

**Affiliations:** Biochemistry Department, College of Science, King Saud University, Riyadh 11495, Saudi Arabia; 444203417@student.ksu.edu.sa (N.A.); szargar@ksu.edu.sa (S.Z.); aldbass@ksu.edu.sa (A.M.A.)

**Keywords:** environmental toxins, gut barrier, CCl_4_-induced intestinal damage, tight junction proteins

## Abstract

Environmental toxins can impair gut microbiota and increase intestinal permeability, contributing to various health problems. While many such toxins are known to disrupt tight junctions and compromise barrier function, research specifically examining carbon tetrachloride (CCl_4_) as a trigger of intestinal epithelial barrier dysfunction remains limited. In this study, 54 young Western albino male rats, weighing 180–200 g, were randomly assigned to nine experimental groups, each comprising six rats. Group 1 received 1 mL of oral saline and served as a control. Groups 2 and 3 received 0.2 g/kg body weight probiotic and prebiotic, respectively, for four weeks. CCl_4_ (1 mL/kg, i.p.) was administered either at the beginning of day 1 (damage induction; Group 4) or at the end of day 28 (protection assessment; Group 7). Intervention groups received probiotics and prebiotics for 4 weeks after (therapeutic) CCl_4_ exposure on day 1 in Groups 5 and 6, respectively. Groups 8 and 9 received probiotics and prebiotics for 4 weeks before CCl_4_ exposure on day 28, respectively. Quantification of gut bacterial populations, serum levels of Occludin and Zonulin, as biomarkers of intestinal permeability, and histopathological analysis of intestinal tissue were conducted. CCl_4_ induces significant intestinal epithelial barrier dysfunction with marked histopathological alterations. Probiotic treatment was more effective than prebiotics at normalizing serum Zonulin and Occludin levels in CCl_4_-induced intestinal damage. Probiotics restore microbial balance by suppressing the overgrowth of pathogenic organisms, while prebiotics confer partial protection. CCl_4_-induced gut barrier disruption is restored through probiotic supplements by restoring gut microbial balance and normalizing tight junction-associated biomarkers.

## 1. Introduction

The human body contains numerous mucosal epithelial layers that directly interface with the external environment and internal systems [1,2]. Among these, the gastrointestinal (GI) tract has the most extensive surface area exposed to external elements and plays a crucial role in overall health [3,4]. The GI mucosa enables immune sensing and nutrient absorption, while also acting as a selective barrier against harmful pathogens and antigens [5]. This dual function is managed through a complex interaction of structural components and molecular mechanisms that dynamically maintain both barrier integrity and immune balance in the GI. Any delicate structural change or injury to the mucosa disrupts intestinal permeability, which is primarily regulated by epithelial tight junctions [6]. Tight junctions are multi-protein complexes that form seals between neighboring epithelial cells and define the boundary between the apical and basolateral membrane regions [7]. They maintain the intestinal barrier by regulating the permeability of ions, nutrients, and water, thereby sustaining health. Disruption of tight junctions can lead to leaky gut syndrome, a clinical condition associated with increased intestinal permeability, chronic inflammation, and microbial imbalance (dysbiosis) [8,9,10,11]. Managing these conditions primarily involves restoring tight junction integrity, a promising therapeutic approach. However, no clinically approved drugs currently target tight junctions explicitly [12,13]. Numerous studies have shown that a few specific bacterial strains can improve gut barrier integrity, restore microbial diversity, reduce inflammation, and reverse epithelial barrier damage [14,15].

Probiotics play a critical role in various cellular functions, particularly in regulating tight junctions to preserve the integrity of the intestinal barrier [16]. Probiotics alleviate inflammation, reduce obesity and oxidative stress, and improve gut microbiota through colonization and proliferation of beneficial bacterial genera such as *Lactobacillus* and *Bifidobacterium*. *Bifidobacteria* modulate the host immune response by influencing various signaling pathways within intestinal epithelial cells [17]. *Lactobacillus* strains increase the expression levels of the cannabinoid receptor type 1 (CB1), which is associated with the increased expression of tight junction proteins, such as Occludin and Zonulin [18]. Prebiotics serve as nutrients for these beneficial gut bacteria, which are fermented into short-chain fatty acids (SCFAs), such as acetate, propionate, and butyrate, thereby strengthening the intestinal barrier [19,20].

Studies have mentioned Carbon tetrachloride (CCl_4_) as a rapid model for inducing tight junction alteration and barrier dysfunction in rodents [21]. In rats, CCl_4_ can disrupt the tight junction network within 24 h of exposure by disintegrating zonulae and occludens in liver tissue; however, studies have not reported on CCl_4_ as an inducer of intestinal epithelial barrier dysfunction [22]. CCl_4_ is well-known for inducing oxidative stress and inflammation [23]. As a toxin, its primary target is the liver, but it can affect the intestinal mucosa and impair its integrity [24]. It can generate reactive free radicals that damage cellular components and disrupt barrier function, causing histological injury in the intestine [25].

Given the relationship between the gut and overall health, the disruption of the intestinal barrier induced by CCl_4_ in rodent models can provide a valuable framework to investigate the mechanisms underlying gut dysfunction. We hypothesized that CCl_4_ can be used to explore therapeutic interventions aimed at restoring gut integrity. In this study, our main objective was to establish a CCl_4_-induced leaky gut model in rodents. Occludin and Zonulin were evaluated as two key markers of intestinal permeability to validate the model. Recovery was monitored through barrier restoration by treatment with commercially available probiotics and prebiotics. We evaluated both the therapeutic and protective potential of probiotics and prebiotics by administering CCl_4_ at the beginning, followed by treatment to assess recovery after damage, and by providing probiotic and prebiotic supplementation before CCl_4_ exposure to assess their protective effect.

## 2. Materials and Methods

### 2.1. Animals

A total of 54 western albino male rats, approximately ±6 weeks old, weighing 180–200 g were obtained from the Experimental Surgery and Animal Lab at KSU. Animals were randomly placed in ventilated cages each containing 6 rats, under controlled laboratory conditions (temperature 23 °C, humidity 55 ± 5% and day/night 12 h light cycle) with free access to food (AIN-93 G, Grain Silos and Flour Mills organization, Riyadh, Saudi Arabia) and water ad libitum.

All the experimental protocols were duly reviewed and approved by the Institutional Animal Ethics Committee, King Saud University, Riyadh (Ref. No. KSU-SE-2024-14).

All experimental procedures involving animals complied with the applicable laws and regulations in accordance with current guidelines, as well as the principles stated in the National Institutes of Health, USPHS and Guidance for the Care and Use of Laboratory Animals.

### 2.2. Experimental Design

After one week of acclimatization, animals were randomly assigned to nine experimental groups, with 6 rats assigned as follows: Control, Probiotic, Prebiotic, CCl_4_ (day 1), CCl_4_ + Probiotic (therapeutic), CCl_4_ + Prebiotic (therapeutic), CCl_4_ (day 28), Probiotic + CCl_4_ (protective), Prebiotic + CCl_4_ (protective).

Group description with treatment details

Group 1—Control group received 1 mL of oral saline.

Group 2—Probiotic group was orally administered with 0.2 g/kg body weight probiotic (Commercially available Multi-strain *Bifidobacterium* and *Lactobacillus*) daily for four weeks [26].

Group 3—Prebiotic group was orally administered with prebiotic in the form of bee pollen at a dose of 0.2 g/kg daily for four weeks [27]. Bee pollen is recognized for its prebiotic properties, primarily due to high fiber, carbohydrates, and polyphenols which promote the growth of beneficial gut bacteria [28].

Group 4—CCl_4_ (day 1) group received a single intraperitoneal injection of CCl_4_ at a dose of 1 mL/kg body weight; diluted 1:1 (*v*/*v*) in olive oil on day one of the study [29].

Group 5—CCl_4_ + Probiotic (therapeutic) group received a single intraperitoneal injection of CCl_4_ (1 mL/kg body weight; 1:1 *v*/*v* diluted in olive oil) followed by daily oral administration of a probiotic (0.2 g/kg body weight) for four weeks.

Group 6—CCl_4_ + Prebiotic (therapeutic) group rats received a single intraperitoneal injection of CCl_4_ (1 mL/kg body weight; 1:1 *v*/*v* diluted in olive oil), followed by daily oral administration of bee pollen (0.2 g/kg body weight) for four weeks.

Group 7—CCl_4_ (day 28) group received a single intraperitoneal injection of CCl_4_ (1 mL/kg body weight; 1:1 *v*/*v* diluted in olive oil) on day 28 of the study [29].

Group 8—Probiotic + CCl_4_ (protective) group received 0.2 g/kg oral dose of probiotic for four weeks and a single intraperitoneal injection of CCl_4_ (1 mL/kg body weight; 1:1 *v*/*v* diluted in olive oil) on day 28.

Group 9—Prebiotic + CCl_4_ (protective) group received prebiotic in form of bee pollen at a dose of 0.2 g/kg daily for four weeks and a single intraperitoneal injection of CCl_4_ (1 mL/kg body weight; 1:1 *v*/*v* diluted in olive oil) on day 28.

The probiotic used in this study was a commercially available multi-strain formulation from Dr. Formulated Probiotics Fitbiotic^®^, Garden of Life, Palm Beach Gardens, FL, USA). Descriptions of the probiotic formulation species with colony-forming units are summarized in Table S1. With an average of 200 g, each rat received approximately 0.04 g of probiotic powder per day, corresponding to approximately 4.8 × 10^8^ CFU per rat per day. The prebiotic was a 100% natural bee pollen powder (Nutricost^®^, Vineyard, UT, USA). At the administered dose of 0.2 g/kg body weight, each rat received approximately 0.04 g (40 mg) per day.

The CCl_4_ at day 1 group represents a therapeutic design, where probiotics/prebiotics were used to treat CCl_4_ induced intestinal damage or leaky gut. The CCl_4_ at day 28 group represents a protective design, where probiotics/prebiotics were given to prevent or reduce the risk of leaky guts.

On day 29, the rats were euthanized by using compressed H_2_O_2_ gas in cylinders. Without pre-charging the chamber, the animal(s) were placed in the chamber which was introduced with 100% CO_2_ at a fill rate of 30–70% displacement of the chamber volume per minute, added to the existing air in the chamber. This achieved rapid unconsciousness with minimal distress to the animals.

### 2.3. Stool Sample Collection

Stool samples from all the groups were collected on day 28 in sterile tubes to assess microbial changes following CCl_4_ exposure under both acute and recovery conditions. CCl_4_ exposure on day 28 showed the acute microbiota response while day 1 exposure reflected changes during the recovery phase over time. Therapeutic and preventive effects were evaluated through treatment and protection groups, respectively, with the control group serving as a baseline reference. All the samples were stored at −20 °C until microbial analysis. Quantitative estimation of aerobic bacteria in the stool cultures of animals from each group was conducted using the method described by Itoh et al. [30].

#### Microbiota Analysis

Quantitative culture was performed on selective and non-selective media (MacConkey agar (MCA), Sabouraud’s dextrose agar (SDA), Mueller–Hinton agar (MHA) and 5% Sheep blood agar) under aerobic conditions at 37.5 °C for 24–48 h. Colonies were identified by morphology, Gram staining, and biochemical tests. The bacterial and fungal loads were quantified using a semi-quantitative colony-forming unit (CFU) scale for comparative analysis of microbial changes among different experimental groups. (+) = Rare: <10^3^ CFU/g feces, (++) = Few: 10^3^–10^4^ CFU/g feces, (+++) = Moderate: 10^5^–10^6^ CFU/g feces, (++++) = Heavy: >10^6^ CFU/g feces.

### 2.4. Blood Sample Collection: Intestinal Permeability Biomarkers

Blood samples were collected from the dorsal aorta into sterile dry glass centrifuge tubes and allowed to clot at room temperature. The clotted blood samples were then centrifuged at 3000 rpm for 15 min, and the resulting serum was carefully separated and stored at −20 °C until biochemical analysis. Serum levels of Occludin and Zonulin as biomarkers of intestinal permeability (leaky gut) were measured using enzyme-linked immunosorbent assay (ELISA) kits from MyBioSource (San Diego, CA, USA), following the manufacturer’s instructions.

#### 2.4.1. Measurement of Zonulin Concentration

Zonulin levels in serum were measured using a Double Antibody Sandwich ELISA kit (MyBioSource, USA; Cat. No. MBS2606662). The kit’s detection range was 100 ng/mL to 1.56 ng/mL. Sensitivity of 0.5 ng/mL. Due to the reported limitations in the specificity of some ELISA-based Zonulin assays, the results were interpreted together with other findings in this study.

#### 2.4.2. Measurement of Occludin Concentration

Occludin levels in serum were determined using a Sandwich ELISA kit (MyBioSource, USA; Cat. No. MBS761321). The detection range was from 0.156 to 10 ng/mL, with a sensitivity of 0.094 ng/mL.

### 2.5. Intestine Sample Collection for Histopathological Analysis

The intestinal samples were collected and immediately immersed in a 10% formalin solution for histological studies, followed by standard dehydration in an ascending series of ethanol, clearing in xylene, and embedding in paraffin wax. Thick sections of 5–7 μm were cut by rotary microtome and mounted on slides, dried, and stained with conventional hematoxylin and eosin (H&E) stain. Photomicrographs were captured using a Nikon 80i light microscope (Nikon Corporation, Tokyo, Japan).

### 2.6. Statistical Analysis

The results are expressed as the means ± standard deviation (SD). All statistical comparisons between the groups were performed using one-way analysis of variance tests, with the Duncan test for range post hoc test was used for multiple comparisons between groups. Significance was assigned at the level of *p* < 0.05. The positive and negative correlations between all measured variables were determined using Pearson’s correlations. Zonulin and occluding levels were also accessed through receiver operating characteristic (ROC) curve analysis. The area under the curve (AUC), sensitivity, and specificity were calculated to evaluate discriminations between CCl_4_-exposed groups and corresponding treatment or protection groups. All statistical calculations were performed using the computer program SPSS (Statistical Package for Social Science) version 11.0.

## 3. Results

### 3.1. Microbial Changes and Dysbiosis Induced by CCl_4_

The change in microbial growth in different treated groups compared to the control is shown in Table 1. Yeast growth was not detected in the control, probiotic, and prebiotic groups (Groups 1–3), but minimal growth was observed in the CCl_4_ day 1 groups (Group 4). A remarkable increase in yeast colonization was observed in Group 7 (CCl_4_ day 28) and Group 9 (prebiotic + CCl_4_ day 28). Notably, Group 8 (probiotic + CCl_4_ day 28) shows moderate yeast presence, indicating a possible suppressive effect of probiotics on yeast overgrowth. The control group showed moderate growth of Gram-negative and Gram-positive bacteria. Probiotic and prebiotic treatments (Groups 2 and 3) resulted in reduced bacterial loads, while early CCl_4_ exposure (Group 4) showed strong growth. When combined with probiotics or prebiotics (Groups 5 and 6), bacterial growth was reduced to moderate levels. Groups exposed to CCl_4_ on day 28 (Groups 7–9) demonstrated significantly higher Gram-negative and Gram-positive bacterial growth compared to other groups, suggesting that CCl_4_ exposure immediately enhances microbial colonization with increased microbial burden. No growth of lactose-fermenting *Enterobacteriaceae* was observed in Groups 1–6. However, strong *Enterobacteriaceae* growth was seen in Groups 7 and 9, while Group 8 (probiotic pre-treatment) remained negative. These results suggest that probiotics may provide partial protection against CCl_4_-induced *Enterobacteriaceae* colonization, whereas prebiotics alone do not offer similar benefits.

### 3.2. Protective Mechanisms of Probiotics and Prebiotics on Intestinal Barrier Markers (Zonulin and Occludin)

**Figure 1:**
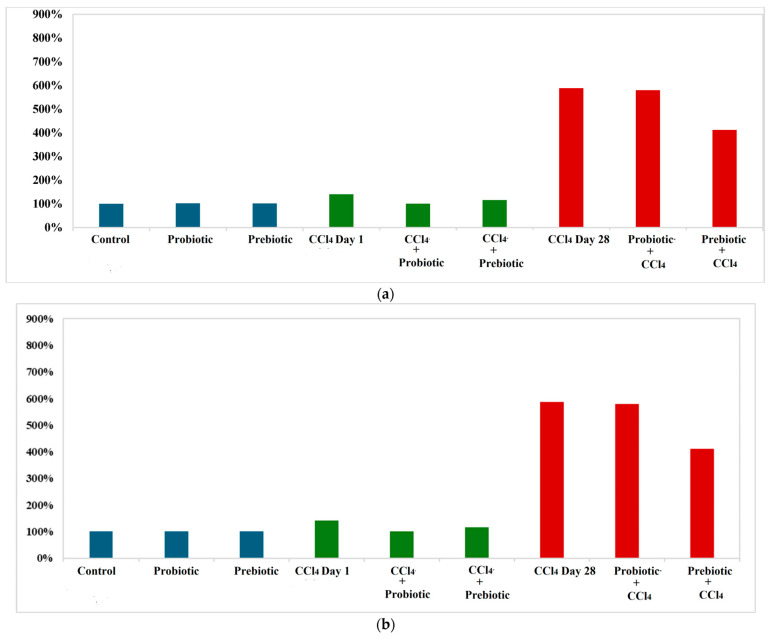
Percent change in mean for different groups according to control group in (**a**) Zonulin and (**b**) Occludin.

**Figure 2:**
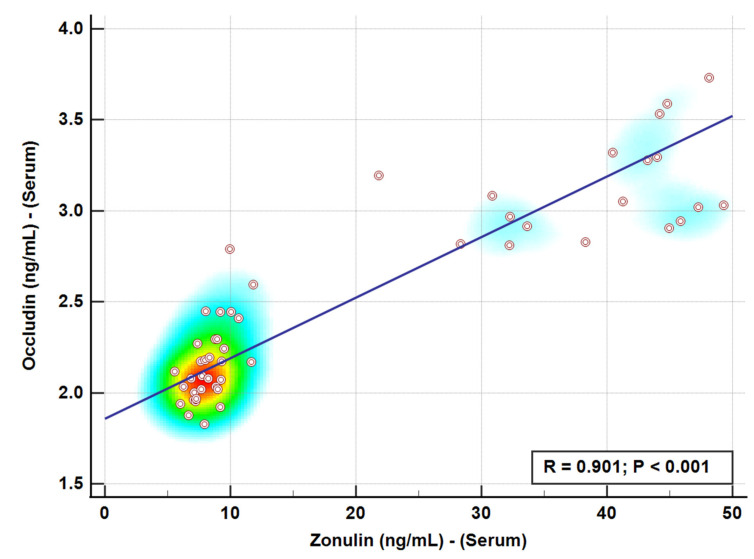
Correlation between Zonulin and Occludin using Person correlation with trend line and heat map (Positive correlation). The color gradient represents the density of data points.

**Figure 3:**
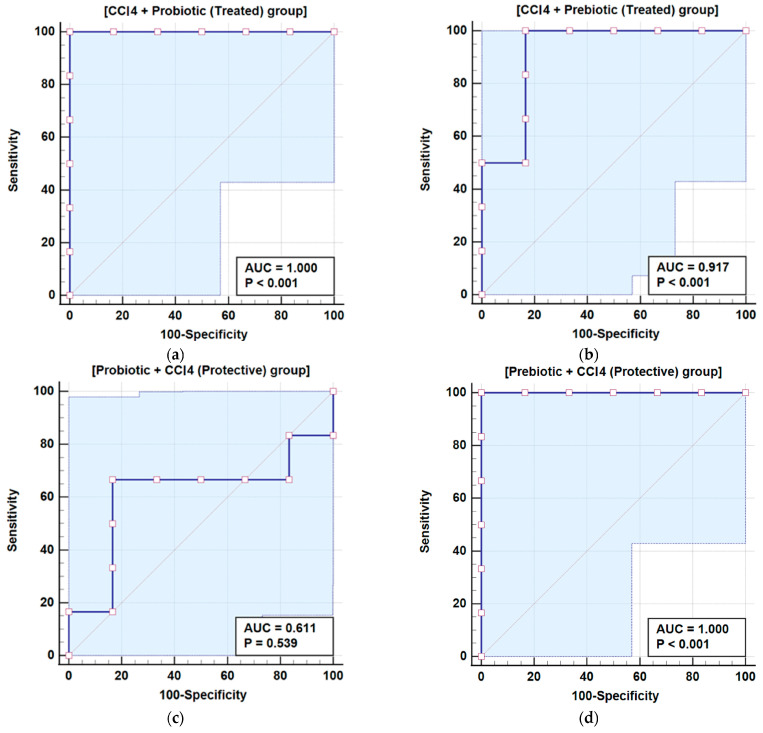
ROC curve for Zonulin with (**a**) CCl_4_ + Probiotic and (**b**) CCl_4_ + Prebiotic with reference to CCl_4_ day 1 group (**c**) Probiotic + CCl_4_ and (**d**) Prebiotic + CCl_4_ with reference to CCl_4_ day 28.

**Figure 4:**
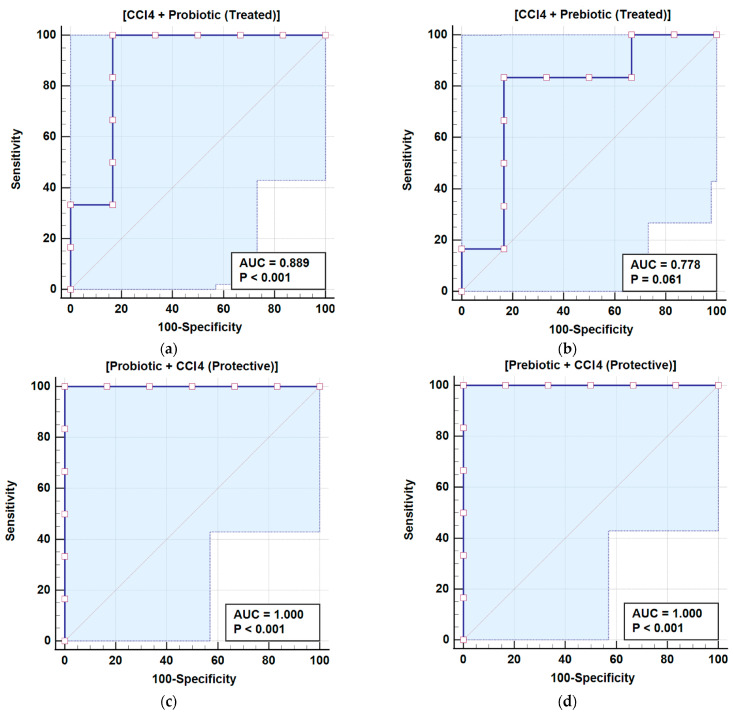
ROC curve for Occludin (**a**) CCl_4_ + Probiotic and (**b**) CCl_4_ + Prebiotic with reference to CCl_4_ day 1 group (**c**) Probiotic + CCl_4_ and (**d**) Prebiotic + CCl_4_ with reference to CCl_4_ day 28 group.

Zonulin and Occludin levels varied significantly across treatment groups. Zonulin levels in the probiotic and prebiotic groups were similar to the control; however, in the CCl_4_ day 1 group, levels were slightly elevated. CCl_4_ day 1, followed by probiotic or prebiotic treatment, reduced Zonulin levels to normal, as shown in Table 2 and Figure 1. Exposure to CCl_4_ on day 28 caused a 588.21% increase in Zonulin levels compared to the control (*p* < 0.001). Similarly, the probiotic + CCl_4_ day 28 group failed to significantly reduce the Zonulin elevation of 579.50%. The prebiotic + CCl_4_ day 28 group showed a 411.38% increase, but levels remained significantly higher than the controls. Occludin levels in the probiotic group decreased significantly by 95.07%, while no notable change was observed in the prebiotic group (99.37%) compared to the control (100%). CCl_4_ exposure on day 1 significantly raised Occludin levels by 121.85%, which decreased with prebiotic (105. 98%) and probiotic (109.51%) treatments, as shown in Groups 5 and 6, respectively, compared to Group 4 with CCl_4_ but no treatment. On day 28, CCl_4_ exposure caused a significant increase in Occludin levels by 170.20%. Both Probiotic + CCl_4_ and Prebiotic + CCl_4_ groups showed substantial increases (147.03% and 144.82%, respectively), though levels were still lower than the CCl_4_ day 28 group, indicating partial modulation (Table 2, Figure 1). A positive correlation between Zonulin and Occludin was observed using Pearson correlation, with a trend line and heat map shown in Figure 2. Receiver operating characteristic (ROC) analysis for Zonulin and Occludin levels is presented in Table 3 and Figure 3 and Figure 4. Zonulin levels in the therapeutic groups showed strong separation between CCl_4_ + Probiotic (AUC = 1.000) and CCl_4_ + Prebiotic (AUC = 0.917), compared with the CCl_4_ alone group. In protective groups with CCl_4_ day 28 as references, Prebiotic + CCl_4_ demonstrated high discrimination (AUC = 1.000), while Probiotic + CCl_4_ showed moderate separation (AUC = 0.611) (Table 3, Figure 3). For Occludin, both Probiotic + CCl_4_ day 28 (AUC = 1.000) and Prebiotic + CCl_4_ day 28 (AUC = 0.889) achieved strong discrimination relative to CCl_4_ alone_,_ whereas CCl_4_ + Prebiotic in the therapeutic group demonstrate moderate discrimination (AUC = 0.778, *p* = 0.061) (Table 3, Figure 4).

### 3.3. Histopathological Evidence of Protection

**Figure 5:**
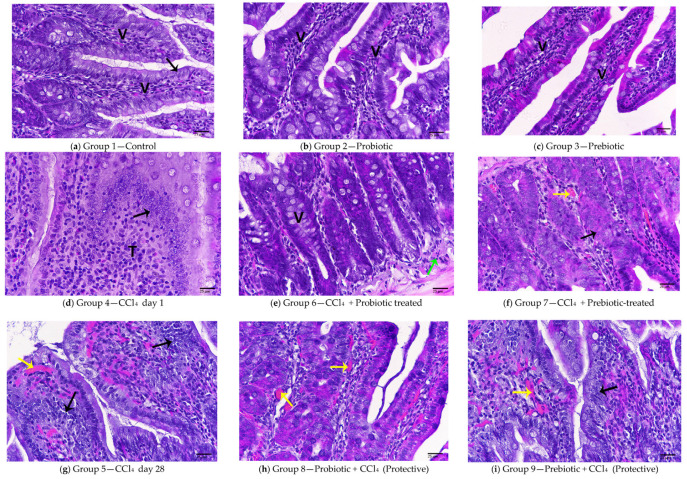
Photomicrograph of (**a**) Control -normal feature without pathological signs, villi (V), columnar epithelia [black arrow]; (**b**) Probiotic group-showing healthy villi; (**c**) Prebiotic group showing healthy villi (V); (**d**) CCl_4_ day 1 group showing displaying tumor node (T), hyperplasia (black arrow); (**e**) CCl_4_ + Probiotic-treated group showing improved villi (V), a few inflammatory cells (green arrow); (**f**) CCl_4_ + Prebiotic-treated group displaying hemorrhage (yellow arrow), hyperplasia (black arrow); (**g**) CCl_4_ day 28 group revealing spacious villi filled with hyperplasia of columnar epithelia (black arrow), ulcer filled with hemorrhage (yellow arrow); (**h**) Probiotic + CCl_4_ protective group revealing marked ulcer filled with hemorrhage (yellow arrow); (**i**) Prebiotic + CCL_4_ Protective group displaying marked ulcer filled with hemorrhage (yellow arrow), hyperplasia (black arrow). (H&E-400×).

The histopathology images of the intestine samples are presented in Figure 5. The control small intestine showing normal villi covered with columnar epithelial cells is shown in Figure 5a. Histological sections of animals treated with probiotics and prebiotics in Figure 5b,c show no pathological features and no detectable pathological alterations. However, animals treated with CCl_4_ on day one exhibited the formation of nodular lesions composed of proliferating epithelial cells intermingled with infiltrating immune cells, as well as abundant hyperplasia of columnar epithelia, which are clearly seen in Figure 5d. The group treated with CCl_4_ followed by probiotics revealed marked improvement manifested by a reduction in pathological signs, the presence of healthy villi, and a reduction in inflammatory cells, as revealed by histological sections in Figure 5e. On the other hand, animals treated with CCl_4_ followed by prebiotic, shown in Figure 5f, revealed some pathological signs, such as hyperplasia and focal hemorrhage within the intestinal mucosa. Animals treated with CCl_4_ 28 days before sacrifice revealed spacious villi with hemorrhage due to ulcers, along with hyperplasia of columnar epithelial lining, shown in Figure 5g. Moreover, the intestine of animals protected with probiotics and then treated with CCl_4_ showed partial amelioration of pathological signs, though ulceration and hemorrhage remained evident, as seen in Figure 5h. Additionally, the intestines of animals protected with prebiotics and then with CCl_4_ demonstrated mild pathological changes, including limited epithelial hyperplasia and minor hemorrhage as shown in Figure 5i.

## 4. Discussion

The intestine is the body’s largest interface with the external environment and a selective barrier that maintains internal stability [9]. The intestinal barrier is actively influenced by gut microbiota and intercellular junctions [15]. Research has highlighted the critical role of tight junction proteins in both normal physiology and gastrointestinal (GI) and liver diseases. TJ proteins maintain barrier integrity within the gut and liver and have the potential to be therapeutic targets for GI diseases [8]. In the present study, CCl_4_ was used to disrupt tight junction networks, and therapeutic strategies aimed at restoring intestinal integrity and gut flora through the use of prebiotics were evaluated. CCl_4_ promotes microbial overgrowth, including yeasts, Gram-negative bacteria, and *Enterobacteriaceae*. Probiotics showed a protective effect, particularly against overgrowth of yeast and *Enterobacteriaceae*. CCl_4_ exposure was associated with disruption of gut microbial homeostasis, as noted immediately after CCl_4_ exposure in Groups 7–9 (Table 1), with a remarkable increase in microbial burden, particularly lactose-fermenting *Enterobacteriaceae*, which were absent in other groups (Groups 1–6). CCl_4_ is reported to disrupt gut flora and induce pathogenic colonization in rodents [31]. Recent studies have reported noticeable gut dysbiosis, including shifts in pathogenic overgrowth in CCl_4_-treated mice [32]. Probiotic yeasts and bacterial strains restore the *Candida* species by competitive inhibition and pH modulation [33]. The protective effect of probiotics in our study is likely due to their antioxidant and anti-inflammatory properties, highlighting their potential as targeted therapies for toxin-induced dysbiosis [34,35]. Zonulin and Occludin were measured as circulating biomarkers of intestinal barrier integrity. Zonulin regulated the intestinal permeability by modulating intercellular tight junctions, and increased serum level reflects enhanced permeability associated with tight junction disassembly. Occludin is a transmembrane tight junction protein expressed in epithelial and endothelial cells that plays a major role in maintaining junctional integrity. Elevated serum Occludin levels are mainly due to the release of junctional components into circulation which indicates epithelial injury or tight junction disruption. The combined assessment of these two biomarkers reflects intestinal barrier integrity in the gut. Probiotics are reported to normalize the intestinal barrier by upregulating tight junction proteins like Occludin and ZO-1 through activation of AMPK, a key mechanism behind assembly and reassembly of tight junctions in epithelial cells [36]. Previous studies suggest that probiotics also inhibits the NF-κB pathway, a major inflammatory signal involved in TJP disruption. Probiotics have also been shown to reduce oxidative stress-induced damage to epithelial junctions by strengthening the barrier and reducing inflammation [37]. In contrast, limited protection of prebiotics supports their inefficiency in managing microbial imbalances due to slower microbial fermentation dynamics or insufficient SCFA production under toxic conditions [38]. SCFAs play a vital role in gut health by supporting the intestinal barrier, reducing inflammation, and inhibiting harmful bacteria. If microbial fermentation of prebiotics is slow or SCFA production is insufficient, the protective effects may be delayed, leaving the gut vulnerable to acute toxin damage [39]. Our results suggest a potential role for probiotics against environmental toxins concerning gut health. The differential effect of probiotics and prebiotics in alleviating CCl_4_-induced alterations on Zonulin and Occludin was timing-dependent. A slight increase in Zonulin after CCl_4_ exposure on day 1 suggests early barrier disruption, which was effectively normalized by both probiotic and prebiotic treatments, indicating a healing potential in acute settings [40]. However, CCl_4_ exposure on day 28 showed significant intestinal injury, showing no protection by probiotic or prebiotic, suggesting a limited capacity of prebiotics to restore barrier function under chronic toxin-induced stress [41]. The positive correlation between Zonulin and Occludin levels suggests that both markers serve as complementary indicators of barrier integrity [42]. ROC analysis further demonstrated the ability of Zonulin and Occludin to discriminate between predefined experimental groups. Intestinal probiotics are critically important for human health [43]. These active microorganisms can renew intestinal epithelial cells, strengthen their connection, and intensify tight junction proteins [44]. Gut probiotics improve the immune system by regulating the intestinal antimicrobial peptides and compete for nutrients and space with pathogenic bacteria to restore the intestinal barrier [45]. In this study, a multi-strain probiotic formula of *Lactobacilli* and *Bifidobacteria* preserves the integrity of the intestinal barrier from damage caused by CCl_4_ by modulating Zonulin or Occludin levels. Our data suggest that probiotics improved tight junction integrity more effectively than prebiotics, although partial protection was observed in protective groups [46]. Probiotics affect gut epithelial integrity by enhancing the expression of tight junction proteins and secreting bioactive compounds, mainly short-chain fatty acids, bacteriocins, and polysaccharide A, to promote epithelial cell proliferation, mucus secretion, and immune tolerance [47]. Probiotics modulate immune responses by downregulating pro-inflammatory cytokines and upregulating anti-inflammatory mediators, reducing inflammation-induced barrier disruption [48]. Prebiotics, on the other hand, act through commensal bacteria present in the gut to generate protective metabolites through fermentation [49].

Altered tight junctions and structural disruption of the epithelial barrier were also confirmed through histopathological observations, which demonstrated clear alterations in intestinal architecture following CCl_4_ exposure. We found differences in protective efficacy between probiotics and prebiotics against CCl_4_-induced intestinal injury. Rats treated with either probiotics or prebiotics alone showed no detectable pathological changes, confirming the safety and non-toxic nature of these interventions under normal physiological conditions [50]. CCl_4_ administration alone resulted in severe intestinal damage. Probiotic treatment markedly ameliorated CCl_4_-induced mucosal injury, restoring villus architecture and reducing inflammation. However, CCl_4_ followed by prebiotics still exhibited signs of mucosal hyperplasia and focal hemorrhage, indicating only partial protection. These findings suggest that prebiotics alone cannot heal the damage caused by CCl_4_, possibly due to their indirect mode of action that depends on gut microbial fermentation [51]. Overall, these results support the efficiency of probiotics over prebiotics in preserving intestinal histology under chemical-induced injury; however, elevated Zonulin levels in the protective groups indicate only partial restoration of barrier function [52].

## 5. Conclusions

The present study suggest that probiotics in the form of multi-strain *Bifidobacterium* and *Lactobacillus* may offer protection against CCl_4_-induced intestinal damage compared to bee pollen-derived prebiotics; however, both interventions were found to be safe under normal physiological conditions. The beneficial outcomes associated with probiotic treatment were associated with improvement in tight junction-related biomarkers and balance by suppressing overgrowth of pathogenic organisms such as *Enterobacteriaceae* and *Candida* spp., as compared to prebiotics which showed partial protection.

## Figures and Tables

**Table 1:** Quantitative assessment of microbial growth among different treated groups compared with control.

Media	Organisms	Group 1 Control	Group 2 Probiotic	Group 3 Prebiotic	Group 4 CCl_4_ Day 1	Group 5 CCl_4_ + Pro	Group 6 CCl_4_ + Pre	Group 7 CCl_4_ Day 28	Group 8 Pro + CCl_4_	Group 9 Pre + CCl_4_
SDA	Yeasts	-	-	-	+	-	-	++++	++	++++
MHA	Gram-negative rods or cocci	++	+	+	+++	+	++	++++	++++	++++
SBA	Gram-positive/Gram-negative rod and cocci	++	+	+	++	++	++	++++	++++	++++
MCA	*Enterobacteriaceae* (Gram-negative rod, lactose fermenters)	-	-	-	-	-	-	++++	-	++++

SDA, sabouraud dextrose agar; Mueller Hinton agar (MHA); Sheep Blood agar (SBA); MacConkey agar (MCA). In cultured plate, bacterial growth is quantified on a scale as follows: - = No growth, less than 10^3^ CFU/gram of feces. + = Rare growth, less than 10^3^ CFU/gram of feces. ++ = Few colonies, 10^3^ to 10^4^ CFU/gram of feces. +++ = Moderate growth, 10^5^ to 10^6^ CFU/gram of feces. ++++ = Heavy growth, more than 10^6^ CFU/gram of feces. For each dilution, colony counts between 30 and 300 are typically used to estimate the culture count.

**Table 2:** Comparison between different groups for Zonulin and Occludin levels compared to control.

Parameters	Groups	Mean ± S.D.	Percent Change	F	*p* Value
Zonulin (ng/mL)	Control	7.50 ± 0.99 a	100.00	152.56	0.001
	Probiotic	7.68 ± 1.01 a	102.35		
	Prebiotic	7.60 ± 1.24 a	101.34		
	CCl_4_ on day 1	10.56 ± 1.04 a	140.74		
	CCl_4_ day 1 + Probiotic	7.58 ± 1.19 a	101.09		
	CCl_4_ day 1 + Prebiotic	8.69 ± 0.84 a	115.86		
	CCl_4_ on day 28	44.13 ± 2.48 c	588.21		
	Probiotic + CCl_4_ day 28	43.47 ± 6.11 c	579.50		
	Prebiotic + CCl_4_ day 28	30.86 ± 5.54 b	411.38		
Occludin (ng/mL)	Control	2.03 ± 0.06 ab	100.00	82.15	0.001
	Probiotic	1.93 ± 0.10 a	95.07		
	Prebiotic	2.02 ± 0.05 ab	99.37		
	CCl_4_ on day 1	2.48 ± 0.21 d	121.85		
	CCl_4_ day 1 + Probiotic	2.15 ± 0.11 bc	105.98		
	CCl_4_ day 1 + Prebiotic	2.22 ± 0.17 c	109.51		
	CCl_4_ on day 28	3.46 ± 0.19 f	170.20		
	Probiotic + CCl_4_ day 28	2.99 ± 0.06 e	147.03		
	Prebiotic + CCl_4_ day 28	2.94 ± 0.16 e	144.82		

Table describes a One-way ANOVA Test between different groups with Multiple Comparisons (Duncan test) within the entire groups. The groups which have different letters are significantly different with each other at significance level (0.05). The groups which have the same letter are not significantly different from each other.

**Table 3:** ROC results for Zonulin and Occludin according to CCl_4_ day 1 and CCl_4_ day 28 as a reference group.

Parameter	Ref. Group	Groups	AUC	Cut-Off Value	Sensitivity %	Specificity %	*p* Value
Zonulin (ng/mL)	CCl_4_ day 1	CCl_4_ + Probiotic	1.000	9.067	100.0%	100.0%	0.001
	CCl_4_ day 1	CCl_4_ + Prebiotic	0.917	9.721	100.0%	83.3%	0.001
	CCl_4_ day 28	Probiotic + CCl_4_	0.611	44.859	66.7%	83.3%	0.539
	CCl_4_ day 28	Prebiotic + CCl_4_	1.000	39.360	100.0%	100.0%	0.001
Occludin (ng/mL)	CCl_4_ day 1	CCl_4_ + Probiotic	0.889	2.351	100.0%	83.3%	0.001
	CCl_4_ day 1	CCl_4_ + Prebiotic	0.778	2.352	83.3%	83.3%	0.061
	CCl_4_ day 28	Probiotic + CCl_4_	1.000	3.165	100.0%	100.0%	0.001
	CCl_4_ day 28	Prebiotic + CCl_4_	1.000	3.237	100.0%	100.0%	0.001

## Data Availability

The original contributions presented in this study are included in the article. Further inquiries can be directed to the corresponding authors.

## Supplementary Materials

The following supporting information can be downloaded at: https://www.mdpi.com/article/10.3390/cimb48030310/s1.
