# Supplementary material for: Multi-Strain Probiotic and Bee Pollen Supplementation Attenuates CCl4-Induced Altered Intestinal Tight Junctions in Rodents
Source: Curr Issues Mol Biol. 2026 Mar 13;48(3):310. doi: 10.3390/cimb48030310 (PMC13025479; doi:10.3390/cimb48030310)
Supplement: Supplementary file 1 [file cimb-48-00310-s001.zip › cimb-4187755-supplementary.pdf]

Table S1. Description of the probiotic formulation.

Probiotic Species		Colony-Forming Units (CFU)
Lactobacillus Species	*Lactobacillus gasseri*	Total 10 Billion CFU
	Lactobacillus acidophilus	
	Lactobacillus rhamnosus	
	Lactobacillus brevis	
	Lactobacillus casei	
	Lactobacillus paracasei	
	Lactobacillus plantarum	
	Lactobacillus salivarius	
	Lactobacillus bulgaricus	
Bifidobacterium Species	*Bifidobacterium lactis*	Total 40 Billion CFU
	Bifidobacterium bifidum	
	Bifidobacterium breve	
	Bifidobacterium infantis	
	Bifidobacterium longum	
Total probiotic culture		50 Billion CFU/4.2 g
